# Association of gamma-glutamyl transferase variability with risk of osteoporotic fractures: A nationwide cohort study

**DOI:** 10.1371/journal.pone.0277452

**Published:** 2023-06-02

**Authors:** Dongyeop Kim, Jee Hyun Kim, Heajung Lee, Iksun Hong, Yoonkyung Chang, Tae-Jin Song

**Affiliations:** 1 Department of Neurology, Seoul Hospital, Ewha Womans University College of Medicine, Seoul, Republic of Korea; 2 Department of Neurology, Mokdong Hospital, Ewha Womans University College of Medicine, Seoul, Republic of Korea; Sohag University Faculty of Medicine, EGYPT

## Abstract

**Objectives:**

Gamma-glutamyl transferase (GGT) is related to inflammation, osteoporosis, and vascular diseases. Recently, changes in metabolic parameters have been proposed as osteoporosis biomarkers. We aimed to assess longitudinally the association of GGT variability with osteoporotic fractures.

**Methods:**

From the National Health Insurance Service-Health Screening Cohort database, participants who underwent three or more health examinations between 2003 and 2008 were included (n = 1,072,432). Variability indexes were as follows: (1) coefficient of variation (CV), (2) standard deviation (SD), and (3) variability independent of the mean (VIM). The primary outcome was occurrence of osteoporotic fracture, defined as identification of one of the following international classification of diseases-10 codes: vertebral fractures (S22.0, S22.1, S32.0, S32.7, T08, M48.4, M48.5, M49.5), hip fractures (S72.0, S72.1), distal radius fractures (S52.5, S52.6), or humerus fractures (S42.2, S42.3).

**Results:**

During a median of 12.3 years (interquartile range 12.1–12.6), osteoporotic fractures occurred in 49,677 (4.6%) participants. In multivariable analysis, GGT variability based on CV positively correlated with the occurrence of osteoporotic fracture (adjusted hazard ratio [HR] of the highest quartile compared with the lowest quartile 1.15, 95% confidence interval [CI] 1.12–1.18, *P* < 0.001). These results were consistent even when GGT variability was defined by SD (adjusted HR 1.22, 95% CI 1.19–1.25, *P* < 0.001) and VIM (adjusted HR 1.12, 95% CI 1.09–1.15, *P* < 0.001).

**Conclusions:**

Increased GGT variability is associated with an increased risk of osteoporotic fractures in the Korean population. Maintaining constant and stable GGT level may help reduce the risk of osteoporotic fractures.

## Introduction

Osteoporotic fractures are a common but serious health issue in humans. In particular, the incidence of osteoporotic fractures increases with age and has become a pressing issue worldwide due to the transition to an aging society [1]. Osteoporotic fractures can cause disability due to the disease itself, and the accompanying economic burden is a significant social problem with costs associated with hospitalization, rehabilitation, and reduced quality of life [2]. Among the various sites of osteoporotic fractures, hip and spine fractures are among the most severe and can cause mortality [3]. Risk factors of osteoporotic fracture include low body weight, premature menopause, smoking, alcohol, physical inactivity, steroid use, and vitamin D insufficiency. In this setting, information on correctable or preventable risk factors for osteoporotic fractures is needed [4].

Gamma-glutamyl transferase (GGT) is one of the representative biomarkers of liver disease. Recently, it has been reported that GGT level is also associated with other diseases including cardiovascular disease, osteoporosis, and decreased bone mineral density (BMD), as well as with mortality [5–7]. To confirm the association between a specific biomarker and disease risk, it may be more reliable to determine whether variability in multiple measurements is more highly associated with risk than is change in only one measurement. In this context, GGT variability is closely related to risk of myocardial infarction, stroke, and heart failure [8, 9].

In previous studies, increased blood GGT level has been associated with osteoporosis and decreased BMD [7, 10]. These results support the possibility that GGT is involved in bone metabolism and may be associated with osteoporosis and related fractures. Moreover, variability in metabolic parameters, including GGT, is closely related to inflammation-associated metabolism and dysregulation of homeostasis, which may be associated with cardiovascular diseases and osteoporosis [9, 11]. However, the association between long-term variation in GGT and osteoporotic fractures remains to be elucidated. Our hypothesis is that increased GGT variability is associated with increased risk of osteoporotic fractures, and we used a nationwide cohort database with a longitudinal setting to investigate the association between GGT variability and osteoporotic fractures.

## Methods

### Data source

The National Health Insurance Service (NHIS) is a single insurer managed by the Korean government, covering nearly 97% of the population, with the remaining 3% covered by the Medical Aid program [12]. The NHIS subscribers are encouraged to undergo a standardized health checkup every year, from which a national health screening database has been established (NHIS-HEALS). The NIHS-HEALS cohort consists of a stratified random sample representing approximately 1,236,000 people between the ages of 40–79 years who underwent health screenings, representing 25% of the total population (dataset number: NHIS-2021-01-715) [13]. This database contains information on the individual demographics, socioeconomic status, medical examination information including laboratory test results, and claims information including diagnosis, prescription, and treatment modalities. Demographic data include basic information such as height and weight and surveys about lifestyle, including smoking and alcohol history [14, 15]. The Institutional Review Board of Ewha Womans University College of Medicine (SEUMC 2022–07–053) approved this study with a waiver of informed consent because the study used deidentified retrospective cohort data.

### Study population

**Fig 1:**
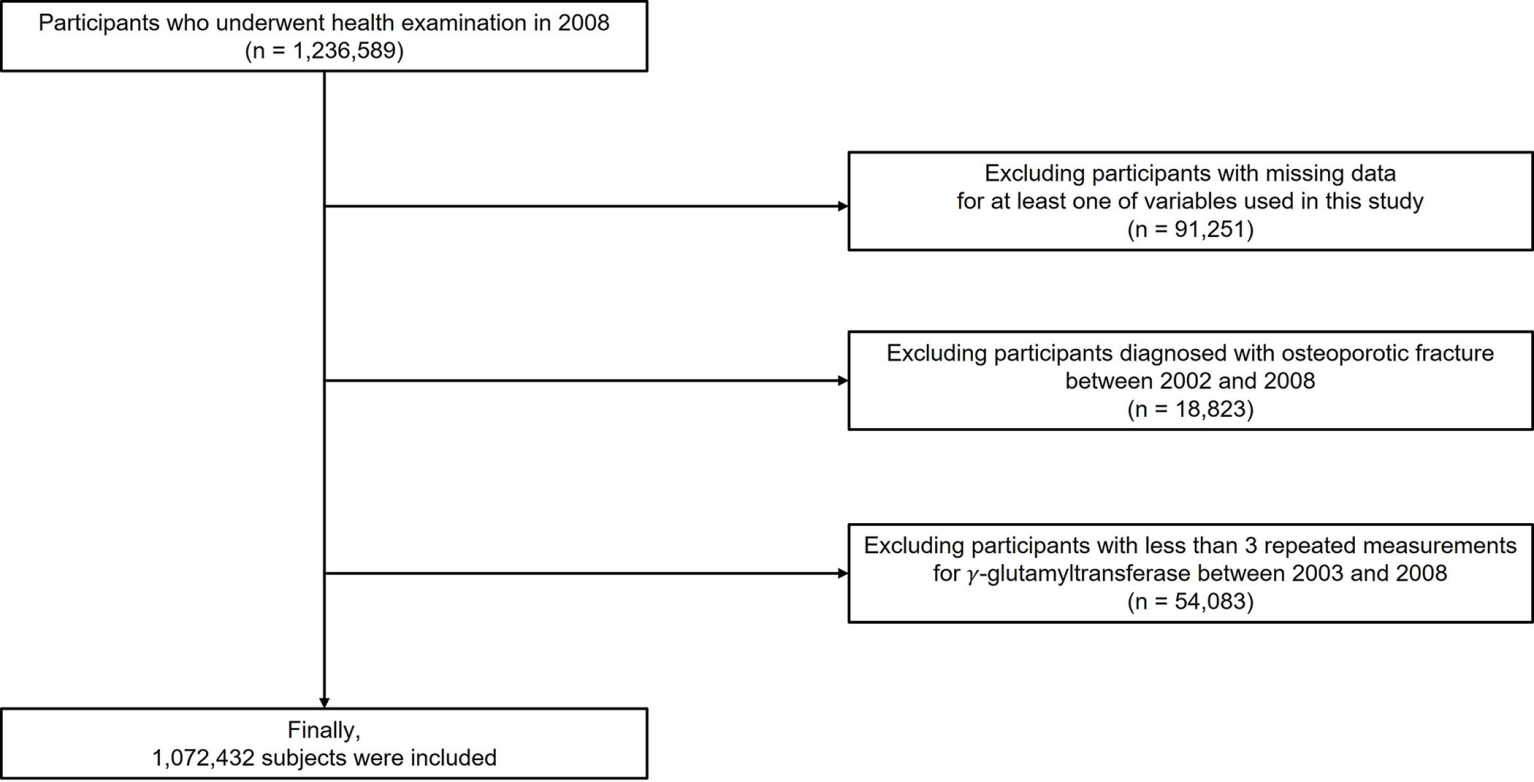
Flowchart of participant enrollment.

Participants from the NHIS-HEALS database who underwent health checkups between 2003 and 2008 were included (n = 1,236,589). Those with missing data on at least one variable of interest (n = 91,251) were excluded. Moreover, participants with a previous history of osteoporotic fracture from January 2002 to the date of checkup (n = 18,823) and participants with fewer than three repeated GGT measurements (n = 54,083) were also excluded. Finally, the data from 1,072,432 people were included and analyzed in this study (Fig 1).

### Definition and variables

The index date was determined as the day of the health checkup in 2008, on which the following baseline characteristics were collected: age, sex, body mass index (BMI), and household income. Information on smoking habits (none, former, and current), alcohol consumption (frequency per week), and regular exercise (frequency per week) was obtained by questionnaires. Comorbidities were defined according to the following criteria between January 2002 and the index date. Hypertension was defined as satisfying one of the following criteria: 1) at least one claim of associated diagnostic codes (International Classification of Diseases, Tenth Revision (ICD)-10 I10–15) with prescription of an antihypertensive agent, 2) two or more claims of diagnostic codes ICD-10 I10–15, 3) systolic/diastolic blood pressure ≥ 140/90 mmHg, or 4) self-reported hypertension in the questionnaire. Diabetes mellitus was defined as satisfying one of the following criteria: 1) at least one claim of related diagnostic codes (ICD-10 E11–14) with prescription of an antidiabetic agent, 2) two or more claims of diagnostic codes ICD-10 E11–14, 3) fasting serum glucose level ≥ 7.0 mmol/L, or 4) self-reported diabetes mellitus in the questionnaire. Dyslipidemia was defined as satisfying one of the following criteria: 1) at least one claim of diagnostic codes (ICD-10 E78) with prescription of a dyslipidemia-related agent, 2) two or more claims of diagnostic codes (ICD-10 E78), and 3) total cholesterol ≥ 240 mg/dL. Stroke was defined as two or more claims of diagnostic code (ICD-10 I60–64). Atrial fibrillation was defined as two or more claims of diagnostic code (ICD-10 I48). Renal disease was defined as two or more claims of diagnostic codes (ICD 10 N17-19, I12-13, E082, E102, E112, E132) or estimated glomerular filtration rate less than 60 mL/min/1.73m^2^. Cancer was defined as at least one admission or at least three outpatient claims of diagnostic code (ICD-10 C00–97) with a specific registration code of ‘V027’ or ‘V193–4’ [16–19].

### Definition of GGT variability

GGT variability was defined as intraindividual change in GGT value in data of health checkups conducted in the 6 years before the index year (2009). Blood tests after at least 8 hours of fasting were performed in laboratories certified by the government, and the results were registered in the national database. Variability indexes were as follows: (1) coefficient of variation (CV), (2) standard deviation (SD), and (3) variability independent of the mean (VIM). The VIM was calculated as 100 x SD/Mean^beta^, where beta is the regression coefficient, on the basis of the natural logarithm of the SD over the natural logarithm of the mean [20].

### Study outcomes

The primary outcome was occurrence of osteoporotic fractures, defined as two or more claims of one of the following ICD-10 codes: 1) vertebral fractures (S22.0, S22.1, S32.0, S32.7, T08, M48.4, M48.5, M49.5), 2) hip fractures (S72.0, S72.1), distal radius fractures (S52.5, S52.6), or humerus fractures (S42.2, S42.3) based on the osteoporotic fracture fact sheet from the Korean Society of Bone and Mineral Research [3]. The follow-up period was calculated as the time between the index date and the time of osteoporotic fractures, the death of the subject, or December 2020, whichever occurred first.

### Statistical analysis

Categorical variables were presented as numbers (percentages) and continuous variables as means ± SDs. For comparisons among groups, categorical variables were analyzed using the chi-square test and continuous variables were analyzed using the analysis of variance test. Restricted cubic splines were assessed to confirm the possibility of a non-linear association between GGT variability and osteoporotic fracture risk, and all GGT variability showed a positive linear association [21]. Kaplan-Meier survival curves were used to investigate the association between quartile of GGT variability and risk of osteoporotic fracture based on the log-rank test. To estimate the incidence of osteoporotic fractures, the total number of events was divided by the sum of person-years. To determine the risk of fracture according to quartile of GGT variability, Cox proportional hazard regression analysis was used, and hazard ratio (HR) with 95% confidence interval (CI) were presented. The multivariable regression model was performed with adjustment for age, sex, BMI, household income, alcohol consumption, smoking status, regular physical activity, and comorbidities (hypertension, diabetes mellitus, dyslipidemia, stroke, atrial fibrillation, cancer, and renal disease). The assumption of the proportionality of hazards was tested using Shoenfeld’s residuals. No departure from the proportional hazards assumption was detected. Subgroup analysis was performed with each kind of osteoporotic fracture (vertebral, hip, distal radius, and humerus fractures). For sensitivity analysis, (1) the mean GGT level was further adjusted in multivariable analysis, and (2) participants with osteoporotic fractures within 1 year from the index date were excluded to minimize the possibility of reverse causality. Cox regression analyses for interactions between osteoporotic fractures and the subgroups depending on covariates were performed among the quartiles of GGT variability based on the CV. Statistical Analysis System software (SAS version 9.2, SAS Institute, Cary, NC) was used for statistical analyses, and a *P*-value < 0.05 was considered to be statistically significant.

## Results

During the median of 12.3 years (interquartile range 12.1–12.6), osteoporotic fractures occurred in 49,677 (4.6%) patients. Regarding the sites of fracture, 24,190 participants had vertebral fractures, 3,843 had hip fractures, 21,191 had distal radius fractures, and 3,910 had humerus fractures. GGT level was measured a total of 5,647,306 times in this study (3 times in 111,538 participants, 4 times in 131,344, 5 times in 189,984, and 6 times in 639,566). Table 1 demonstrates the results of comparative analysis of the groups according to quartile of GGT variability based on CV. Participants with higher quartile of GGT variability were more likely to be men and older and had higher frequencies of hypertension, diabetes mellitus, dyslipidemia, stroke, atrial fibrillation, renal disease, and cancer.

**Table 1:** Baseline characteristics of subjects according to gamma-glutamyl transferase variability.

Variable	Total	Q1	Q2	Q3	Q4	*P*-value
Number of participants (%)	1072432	268078 (25.0)	268138 (25.0)	268108 (25.0)	268108 (25.0)	
Age, years	43.71±10.05	43.72±10.17	43.53±9.85	43.49±9.87	44.13±10.29	< .001
Sex						< .001
Male	828272 (77.2)	196733 (73.4)	206802 (77.1)	211617 (78.9)	213120 (79.5)	
Female	244160 (22.8)	71345 (26.6)	61336 (22.9)	56491 (21.1)	54988 (20.5)	
Body mass index (kg/m^2^)	23.77±3.02	23.27±2.97	23.67±2.99	23.97±3.00	24.16±3.02	< .001
Household income						< .001
Q1, lowest	155494 (14.5)	39482 (14.7)	36473 (13.6)	36904 (13.8)	42635 (15.9)	
Q2	334651 (31.2)	83523 (31.2)	81188 (30.3)	82429 (30.7)	87511 (32.6)	
Q3	392146 (36.6)	96297 (35.9)	100120 (37.3)	100084 (37.3)	95645 (35.7)	
Q4, highest	190141 (17.7)	48776 (18.2)	50357 (18.8)	48691 (18.2)	42317 (15.8)	
Smoking status						< .001
Never	549519 (51.2)	149146 (55.6)	138848 (51.8)	132412 (49.4)	129113 (48.2)	
Former	159648 (14.9)	37303 (13.9)	40208 (15.0)	40961 (15.3)	41176 (15.4)	
Current	363265 (33.9)	81629 (30.5)	89082 (33.2)	94735 (35.3)	97819 (36.5)	
Alcohol consumption (days/week)						< .001
None	667082 (62.2)	183677 (68.5)	171397 (63.9)	160841 (60.0)	151167 (56.4)	
1–4	386503 (36.0)	81411 (30.4)	93046 (34.7)	102534 (38.2)	109512 (40.9)	
≥ 5	18847 (1.8)	2990 (1.1)	3695 (1.4)	4733 (1.8)	7429 (2.8)	
Regular physical activity (days/week)						< .001
None	461467 (43.0)	118316 (44.1)	114913 (42.9)	113712 (42.4)	114526 (42.7)	
1–4	543876 (50.7)	133784 (49.9)	136839 (51.0)	137684 (51.4)	135569 (50.6)	
≥ 5	67089 (6.3)	15978 (6.0)	16386 (6.1)	16712 (6.2)	18013 (6.7)	
Comorbidities						
Hypertension	210665 (19.6)	43704 (16.3)	48487 (18.1)	53720 (20.0)	64754 (24.2)	< .001
Diabetes mellitus	106262 (9.9)	20469 (7.6)	23043 (8.6)	26972 (10.1)	35778 (13.3)	< .001
Dyslipidemia	207890 (19.4)	41836 (15.6)	47363 (17.7)	53532 (20.0)	65159 (24.3)	< .001
Stroke	10458 (1.0)	2102 (0.8)	2212 (0.8)	2514 (0.9)	3630 (1.4)	< .001
Atrial fibrillation	4202 (0.4)	822 (0.3)	860 (0.3)	951 (0.4)	1569 (0.6)	< .001
Renal disease	12679 (1.2)	2459 (0.9)	2726 (1.0)	3167 (1.2)	4327 (1.6)	< .001
Cancer	23611 (2.2)	5027 (1.9)	5129 (1.9)	5516 (2.1)	7939 (3.0)	< .001
Aspartate aminotransferase (U/L)	25.14±12.41	22.94±9.08	24.02±10.24	25.41±15.77	29.82±32.65	< .0001
Alanine aminotransferase (U/L)	26.12±17.42	22.35±13.79	24.83±15.85	27.60±19.91	34.10±41.54	< .0001
Mean gamma-glutamyl transferase (U/L)	37.97±37.94	27.10±23.29	32.23±28.82	38.54±35.08	54.00±52.40	< .001
Gamma-glutamyl transferase variability						
CV (%)	26.85±16.15	11.85±3.20	19.83±2.04	27.83±2.77	47.91±17.38	< .001
SD	12.00±20.61	3.26±3.09	6.42±5.88	10.79±10.07	27.53±34.69	< .001
VIM (%)	10.51±5.86	4.93±1.39	8.08±1.14	11.06±1.61	17.97±6.20	< .001

P-value by Chi-square test. Data are expressed as the mean ± SD, or n (%).

Q, quartile; CV, coefficient of variation; SD, standard deviation; VIM, variability independent of the mean.

**Fig 2:**
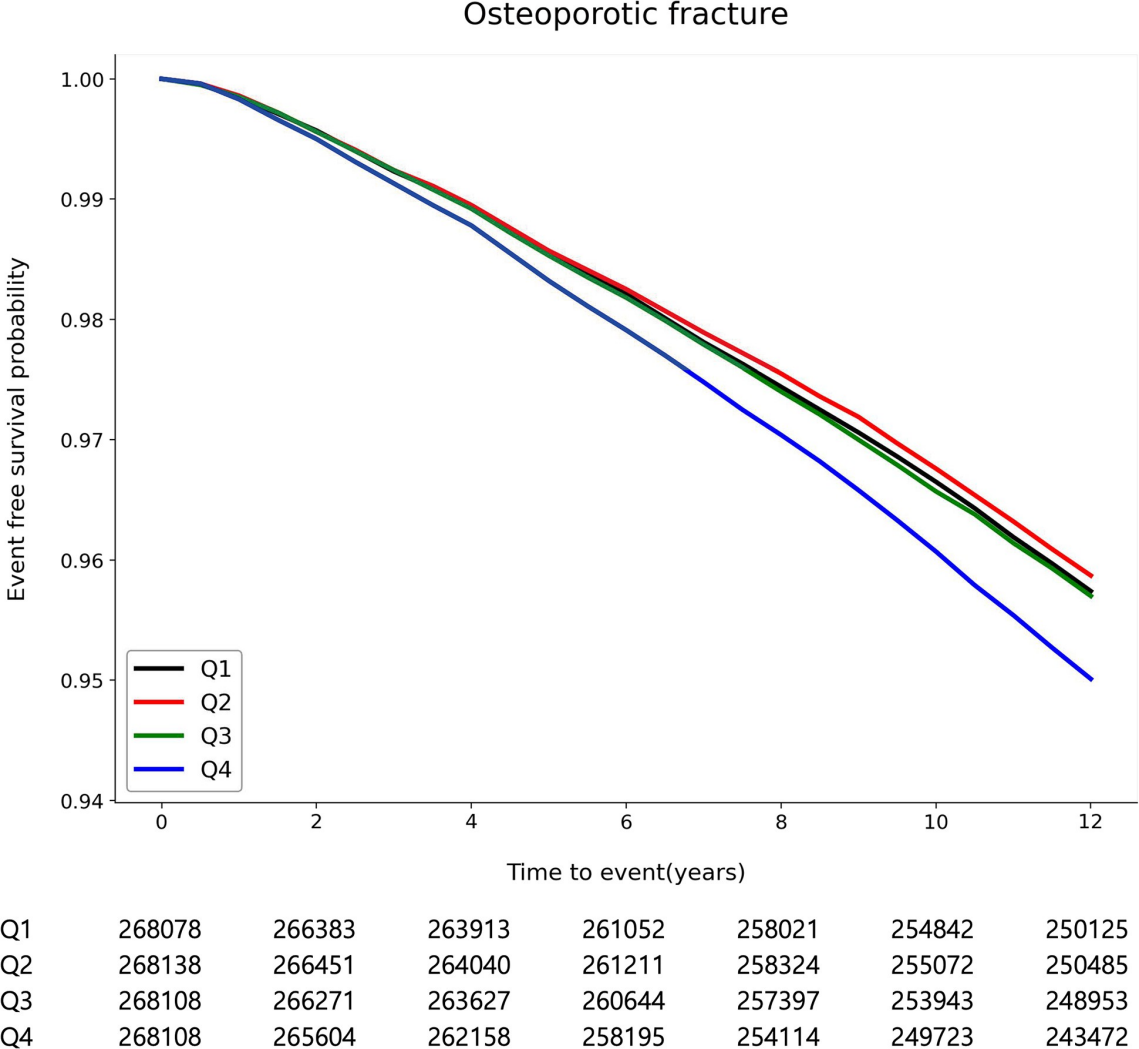
Kaplan-Meier survival curves for incident osteoporotic fracture according to gamma-glutamyl transferase variability based on the coefficient of variation Q, quartile.

Kaplan-Meier survival curves of freedom from osteoporotic fracture are shown in Fig 2 according to GGT variability based on CV. The risk for incident osteoporotic fracture was higher in higher quartiles of GGT variability (*P* < 0.001). In multivariable analysis, quartile of GGT variability based on CV positively correlated with the occurrence of osteoporotic fracture (adjusted HR [highest quartile compared with the lowest quartile] 1.15, 95% CI 1.12–1.18, *P* < 0.001). This trend was consistent even when GGT variability was applied to SD (adjusted HR [highest quartile compared with the lowest quartile] 1.22, 95% CI 1.19–1.25, *P* < 0.001) and VIM (adjusted HR [highest quartile compared with the lowest quartile] 1.12, 95% CI 1.09–1.15, *P* < 0.001), and the results were consistent after adjustment of mean GGT level (Table 2, S1 Table). When divided into deciles of GGT variability for any parameter, the risk of fracture was significantly higher in the highest four deciles compared to the lowest decile, and a significant trend was confirmed with an increase in the risk of fracture as decile increased (S2 Table). In addition, the association between GGT variability and osteoporotic fracture was consistently observed in landmark analysis using a start time of 1 year after the index date (S3 Table).

**Table 2:** The risk for the occurrence of osteoporotic fractures according to quartiles of gamma-glutamyl transferase variability.

						Multivariable model (1)			Multivariable model (2)		
	Number of participants	Number of events	Event rate (%) (95% CI)	Person-years	Incidence rate (per 1000 person-years)	Adjusted HR (95% CI)	*P*-value	*P*-value for trend	Adjusted HR (95% CI)	*P*-value	*P*-value for trend
CV								< .001			< .001
Q1	268078	11975	4.47 (4.39, 4.55)	3228757.01	3.71	1 (reference)			1 (reference)		
Q2	268138	11638	4.34 (4.26, 4.42)	3230996.28	3.60	1.02 (1.00, 1.05)	0.099		1.02 (0.99, 1.04)	0.203	
Q3	268108	12086	4.51 (4.43, 4.59)	3221250.67	3.75	1.07 (1.04, 1.10)	< .001		1.06 (1.03, 1.08)	< .001	
Q4	268108	13978	5.21 (5.13, 5.30)	3186952.90	4.39	1.15 (1.12, 1.18)	< .001		1.10 (1.07, 1.13)	< .001	
SD								< .001			< .001
Q1	268562	13026	4.85 (4.77, 4.93)	3236931.50	4.02	1 (reference)			1 (reference)		
Q2	267563	12272	4.59 (4.51, 4.67)	3220422.54	3.81	1.02 (1.00, 1.05)	0.096		1.02 (0.99, 1.04)	0.185	
Q3	268213	11788	4.40 (4.32, 4.47)	3222799.17	3.66	1.08 (1.05, 1.11)	< .001		1.06 (1.03, 1.09)	< .001	
Q4	268094	12591	4.70 (4.61, 4.78)	3187803.65	3.95	1.22 (1.19, 1.25)	< .001		1.11 (1.08, 1.15)	< .001	
VIM								< .001			< .001
Q1	268108	11842	4.42 (4.34, 4.50)	3226685.03	3.67	1 (reference)			1 (reference)		
Q2	268119	11445	4.27 (4.19, 4.35)	3229533.56	3.54	1.01 (0.99, 1.04)	0.292		1.01 (0.99, 1.04)	0.410	
Q3	268136	12076	4.50 (4.42, 4.58)	3221272.46	3.75	1.06 (1.03, 1.09)	< .001		1.05 (1.02, 1.08)	< .001	
Q4	268069	14314	5.34 (5.25, 5.43)	3190465.82	4.49	1.12 (1.09, 1.15)	< .001		1.10 (1.07, 1.13)	< .001	

Multivariable model (1) was adjusted for age, sex, body mass index, income levels, smoking, alcohol consumption, regular physical activity, hypertension, diabetes mellitus, dyslipidemia, stroke, atrial fibrillation, renal disease, cancer, aspartate aminotransferase, and alanine aminotransferase levels.

Multivariable model (2) was adjusted for age, sex, body mass index, income levels, smoking, alcohol consumption, regular physical activity, hypertension, diabetes mellitus, dyslipidemia, stroke, atrial fibrillation, renal disease, cancer, aspartate aminotransferase, alanine aminotransferase, and mean gamma-glutamyl transferase levels.

CI, confidence interval; HR, hazard ratio; CV, coefficient of variation; Q, quartile; SD, standard deviation; VIM, variability independent of the mean.

In subgroup analyses testing *P*-value for interaction, significant interaction effects regarding the presence of osteoporotic fractures were observed for sex and alcohol consumption, with a prominent association in women compared to men (adjusted HR 1.19, 95% CI 1.15–1.23 vs. adjusted HR 1.08, 95% CI 1.04–1.12, *P* = 0.035) and individuals with alcohol use ≥ 5 or 1–4 days/week compared to those with < 1 day/week (adjusted HR 1.28, 95% CI 1.09–1.51 vs. adjusted HR 1.22, 95% CI 1.16–1.28 vs. adjusted HR 1.11, 95% CI 1.08–1.15, *P* = 0.027) (S4 Table).

In further analyses by type of osteoporotic fracture, the third and highest quartiles of GGT variability were significantly associated with risk of vertebral, hip, distal radius, and humerus fractures compared to the lowest quartile of GGT variability regardless of variability parameter except between GGT variability based on VIM and risk of distal radius fracture (S5–S8 Tables, S1 Fig).

## Discussion

The key findings of our study are that high GGT variability is associated with an increased risk of osteoporotic fracture after adjustment of potential confounders, including age, sex, BMI, alcohol consumption, physical exercise, and comorbidities. Moreover, these associations were consistent regardless of the location of fractures (vertebral, hip, distal radius, and humerus fracture) or the variability parameter (CV, SD, and VIM). In subgroup analysis, more prominent association was observed in women than men and in heavy drinkers than non-drinkers. The effect of GGT variability may be emphasized in these subgroups as woman and heavy drinking are significant risk factors for osteoporotic fractures.

Although not a large number of studies has been conducted, results have been consistent regarding serum GGT level and risk of osteoporosis represented by bone mass. A previous single clinic-based cross-sectional study demonstrated that increased GGT level was correlated with decreased BMD level measured using dual-energy X-ray absorptiometry after adjustment of confounding variables [22]. In a population-based cross-sectional study conducted in Korea, GGT level had a more distinct negative relationship with BMD at multiple bony sites compared to other liver enzymes such as aspartate transaminase and alanine transaminase, and its effect persisted after adjustment of age-sex interactions [7].

While serum GGT level is clinically used as a biomarker indicating hepatobiliary injury or chronic alcohol consumption, one of the major physiologic roles of GGT attached to the cell membrane is to metabolize extracellular glutathione and enable the transfer of amino acids into the cell for re-synthesis of glutathione [23]. On the other hand, increased GGT activity contributes to prooxidant activity through free radical formation, lipid peroxidation, and mutagenesis, which occurs at the outer surface of the cell membrane [24]. The prooxidant and proinflammatory activities caused by increased GGT are believed to play a role in the pathogenesis of various human diseases, and GGT has been studied for its efficacy as a predictive biomarker of atherosclerosis, heart failure, metabolic syndrome, non-alcoholic liver diseases, chronic kidney disease, cancers, and fractures [23, 25, 26].

Oxidative stress, which is associated with increased GGT activity, has been extensively studied in terms of bone remodeling and suggested to contribute to pathological changes in mineralized tissue. Reactive oxygen species are involved in osteoclast formation and subsequent bone resorption in mouse marrow culture cells [27] and also in human bone marrow stromal cells via expression of receptor activator of nuclear factor-kappa B ligand (RANKL) and macrophage colony-stimulating factor [28]. Moreover, oxidative stress is suggested to inhibit the differentiation of osteoblasts by activation of extracellular signal-regulated kinase (ERK) and ERK-dependent NF-κB signaling pathways [29]. Decreased BMD at various sites was confirmed to be related to increased oxidative biomarkers in humans [30, 31]. The relationship between GGT and bone homeostasis is thought to be mainly mediated by osteoclastic activation. Extracellular GGT protein induces osteoclastogenesis in mouse bone marrow cells through the expression of RANKL and Toll-like receptor 4 [32, 33]. These findings were confirmed by observation of enhanced bone resorption and subsequent osteoporosis-like conditions in transgenic mice with overexpression of GGT [34].

Several epidemiologic studies have also found a relationship between GGT level and fracture risk, and it seems clear and consistent that there is a positive correlation, in particular with hip fracture risk. A single clinic-based longitudinal study conducted in Korea was the first to report that high baseline serum GGT level in men over 50 years of age may be useful as a biomarker for future osteoporotic fracture risk, regardless of drinking habits [35].

Large population-based cohort studies have also recently confirmed a significant association between GGT level and hip fracture risk [10, 36]. However, there has been no previous study using GGT variability as a biomarker for risk assessment of osteoporotic fractures. The high variability of biomarkers has been attracting attention as a new factor predicting poor clinical outcomes of various diseases, and high GGT variability was identified as a predictor of increased risk of myocardial infarction, stroke, dementia, chronic kidney disease, and mortality [8, 37, 38].

We acknowledge several limitations in this study. First, it should be noted that the study may have been limited by the potential confounding factors that were not considered. The presence of chronic liver diseases, especially those commonly associated with elevated GGT levels, was difficult to determine from this dataset, and therefore could not be adjusted for in the analysis. In addition, we could not consider the precise amount of alcohol and smoking due to the lack of information provided in the dataset. Second, we could not perform a further analysis to distinguish between multiple-site fractures and single-site fractures as our primary endpoint, as it was not possible to determine whether the fractures in multiple locations occurred simultaneously or sequentially when confirming the corresponding ICD-10 codes. Third, since this study was conducted with the Korean population, application to other races or ethnic groups may lead to errors. Fourth, we were unable to establish a clear biological basis or specific cause of GGT variability. Fifth, our retrospective observational study design cannot suggest a causal relationship. On the other hand, there are strengths of this study as well. We used large national representative data with a long follow up to elucidate the effect of GGT variability on osteoporotic fracture occurrence. Our large-scale epidemiologic study provides new perspectives supporting the benefits of maintaining GGT level for prevention of osteoporotic fractures.

## Conclusion

This nationwide population-based cohort study conducted in Korea demonstrated that an increase in GGT variability is associated with an increased risk of osteoporotic fractures, regardless of site. Maintaining stable GGT level may help reduce the risk of osteoporotic fractures.

## Supporting information

S1 Fig Kaplan-Meier survival curves for incident osteoporotic fracture at various sites according to gamma-glutamyl transferase variability based on the coefficient of variation. (A) vertebral fracture, (B) hip fracture, (C) distal radius fracture, and (D) humerus fracture. Q, quartile.
(TIF)

S1 Table Risk factors for the occurrence of osteoporotic fractures. (DOCX)

S2 Table The risk for the occurrence of osteoporotic fractures according to deciles of gamma-glutamyl transferase variability. (DOCX)

S3 Table The risk for the occurrence of osteoporotic fractures according to quartiles of gamma-glutamyl transferase variability, landmark analysis using a start time of 1 year after the index date. (DOCX)

S4 Table The subgroup analysis regarding gamma-glutamyl transferase variability based on the coefficient of variation and osteoporotic fractures in association with demographics or comorbidities. (DOCX)

S5 Table The risk for the occurrence of vertebral fractures according to quartiles of gamma-glutamyl transferase variability. (DOCX)

S6 Table The risk for the occurrence of hip fractures according to quartiles of gamma-glutamyl transferase variability. (DOCX)

S7 Table The risk for the occurrence of distal radius fractures according to quartiles of gamma-glutamyl transferase variability. (DOCX)

S8 Table The risk for the occurrence of humerus fractures according to quartiles of gamma-glutamyl transferase variability. (DOCX)
