# Supplementary figures and images for: Association of gamma-glutamyl transferase variability with risk of osteoporotic fractures: A nationwide cohort study
Source: PLoS One. 2023 Jun 2;18(6):e0277452. doi: 10.1371/journal.pone.0277452 (PMC10237661; doi:10.1371/journal.pone.0277452)

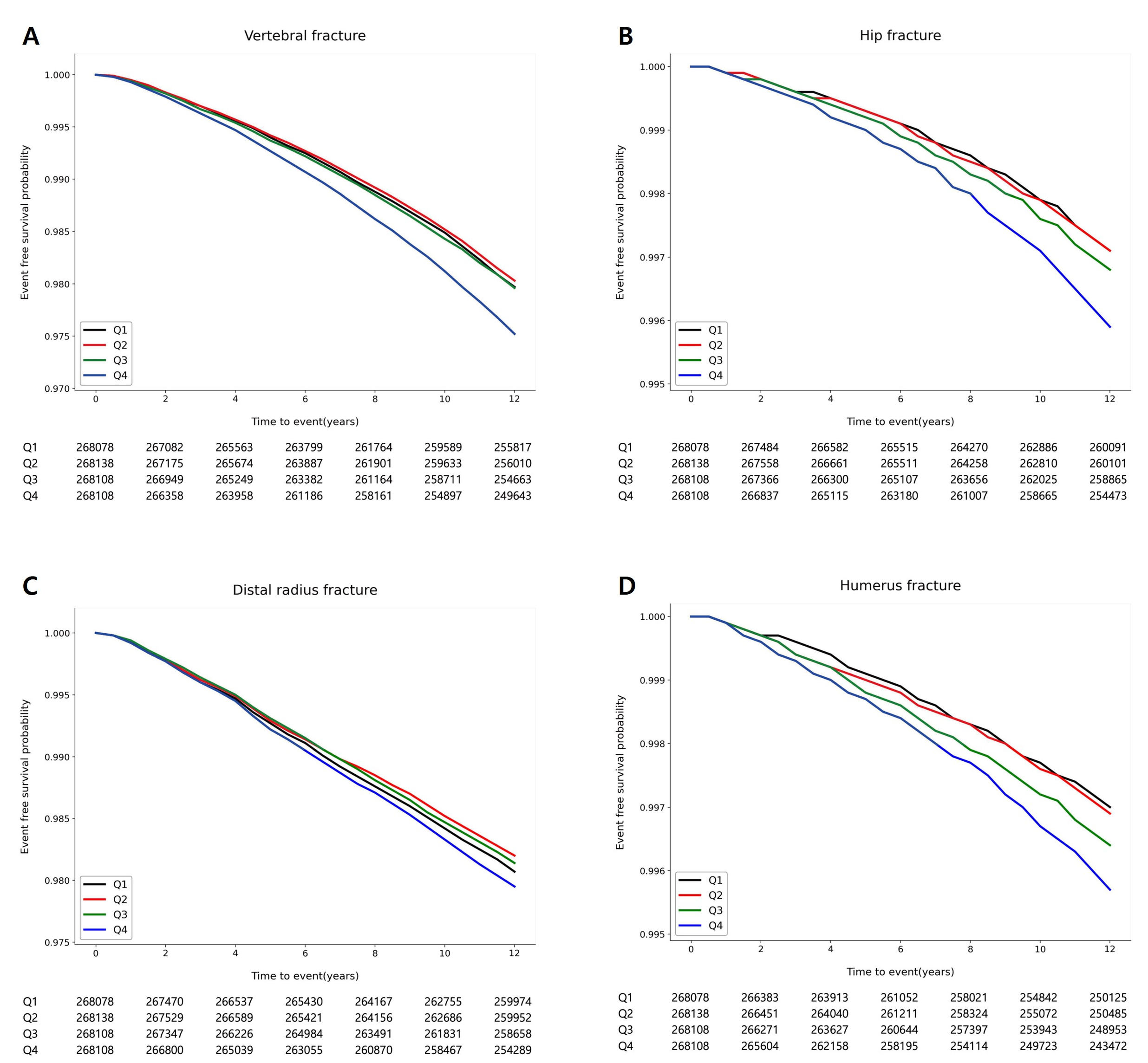

Supplement: S1 Fig — (A) vertebral fracture, (B) hip fracture, (C) distal radius fracture, and (D) humerus fracture. Q, quartile. (TIF) [file pone.0277452.s001.tif]
