# Supplementary material for: Association of gamma-glutamyl transferase variability with risk of osteoporotic fractures: A nationwide cohort study
Source: PLoS One. 2023 Jun 2;18(6):e0277452. doi: 10.1371/journal.pone.0277452 (PMC10237661; doi:10.1371/journal.pone.0277452)
Supplement: S1 Table — (DOCX) [file pone.0277452.s002.docx]

**Supplementary Table 1.** Risk factors for the occurrence of osteoporotic fractures.

|  |  |  | Multivariable model (1) | | Multivariable model (2) | |
| --- | --- | --- | --- | --- | --- | --- |
| Variable | Crude HR (95% CI) | *P*-value | Adjusted HR (95% CI) | *P*-value | Adjusted HR (95% CI) | *P*-value |
| Age, years | 1.08 (1.08, 1.08) | <.001 | 1.07 (1.07, 1.07) | <.001 | 1.07 (1.07, 1.07) | <.001 |
| Sex |  |  |  |  |  |  |
| Male | 1 (reference) |  | 1 (reference) |  | 1 (reference) |  |
| Female | 2.32 (2.28, 2.37) | <.001 | 2.21 (2.15, 2.26) | <.001 | 2.28 (2.23, 2.34) | <.001 |
| Body mass index (kg/m^2^) | 1.00 (1.00, 1.01) | 0.068 | 1.00 (1.00, 1.00) | 0.176 | 1.00 (0.99, 1.00) | 0.006 |
| Household income |  |  |  |  |  |  |
| Q1, lowest | 1 (reference) |  | 1 (reference) |  | 1 (reference) |  |
| Q2 | 0.52 (0.51, 0.54) | <.001 | 1.00 (0.98, 1.03) | 0.973 | 1.00 (0.98, 1.03) | 0.960 |
| Q3 | 0.40 (0.39, 0.41) | <.001 | 0.91 (0.89, 0.94) | <.001 | 0.91 (0.89, 0.94) | <.001 |
| Q4, highest | 0.42 (0.41, 0.43) | <.001 | 0.77 (0.74, 0.79) | <.001 | 0.77 (0.74, 0.79) | <.001 |
| Smoking status |  |  |  |  |  |  |
| Never | 1 (reference) |  | 1 (reference) |  | 1 (reference) |  |
| Former | 0.60 (0.58, 0.62) | <.001 | 0.98 (0.95, 1.01) | 0.218 | 0.98 (0.95, 1.01) | 0.207 |
| Current | 0.60 (0.58, 0.61) | <.001 | 1.16 (1.13, 1.19) | <.001 | 1.14 (1.11, 1.17) | <.001 |
| Alcohol consumption (days/week) |  |  |  |  |  |  |
| None | 1 (reference) |  | 1 (reference) |  | 1 (reference) |  |
| 1-4 | 0.69 (0.68, 0.71) | <.001 | 1.04 (1.01, 1.06) | 0.002 | 1.01 (0.99, 1.03) | 0.418 |
| ≥ 5 | 1.44 (1.36, 1.51) | <.001 | 1.25 (1.18, 1.32) | <.001 | 1.17 (1.11, 1.24) | <.001 |
| Regular physical activity (days/week) |  |  |  |  |  |  |
| None | 1 (reference) |  | 1 (reference) |  | 1 (reference) |  |
| 1-4 | 0.82 (0.80, 0.83) | <.001 | 0.97 (0.95, 0.99) | 0.004 | 0.97 (0.96, 0.99) | 0.006 |
| ≥ 5 | 1.18 (1.14, 1.22) | <.001 | 0.98 (0.95, 1.02) | 0.267 | 0.99 (0.95, 1.02) | 0.438 |
| Comorbidities |  |  |  |  |  |  |
| Hypertension | 1.93 (1.89, 1.97) | <.001 | 0.99 (0.97, 1.01) | 0.342 | 0.98 (0.96, 1.00) | 0.066 |
| Diabetes mellitus | 1.86 (1.82, 1.90) | <.001 | 1.06 (1.04, 1.09) | <.001 | 1.05 (1.02, 1.07) | 0.001 |
| Dyslipidemia | 1.65 (1.62, 1.68) | <.001 | 1.05 (1.03, 1.08) | <.001 | 1.04 (1.02, 1.07) | <.001 |
| Stroke | 2.97 (2.81, 3.14) | <.001 | 1.19 (1.12, 1.25) | <.001 | 1.19 (1.13, 1.26) | <.001 |
| Atrial fibrillation | 2.12 (1.92, 2.34) | <.001 | 1.10 (1.00, 1.22) | 0.052 | 1.10 (0.99, 1.21) | 0.066 |
| Renal disease | 1.99 (1.88, 2.12) | <.001 | 1.06 (1.00, 1.13) | 0.059 | 1.07 (1.01, 1.14) | 0.032 |
| Cancer | 1.93 (1.84, 2.02) | <.001 | 1.16 (1.11, 1.21) | <.001 | 1.16 (1.11, 1.21) | <.001 |
| Aspartate aminotransferase (U/L) | 1.04 (1.01, 1.07) | <.001 | 1.03 (1.01, 1.05) | <.001 | 1.02 (1.01, 1.04) | <.001 |
| Alanine aminotransferase (U/L) | 1.04 (1.01, 1.06) | <.001 | 1.03 (1.01, 1.04) | <.001 | 1.03 (1.01, 1.04) | <.001 |
| Mean gamma-glutamyl transferase (U/L) | 1.03 (1.01, 1.05) | <.001 |  |  | 1.02 (1.01, 1.03) | <.001 |
| Gamma-glutamyl transferase variability |  |  |  |  |  |  |
| Q1 | 1 (reference) |  | 1 (reference) |  | 1 (reference) |  |
| Q2 | 0.97 (0.95, 1.00) | 0.019 | 1.02 (1.00, 1.05) | 0.099 | 1.02 (0.99, 1.04) | 0.203 |
| Q3 | 1.01 (0.99, 1.04) | 0.393 | 1.07 (1.04, 1.10) | <.001 | 1.06 (1.03, 1.08) | <.001 |
| Q4 | 1.18 (1.15, 1.21) | <.001 | 1.15 (1.12, 1.18) | <.001 | 1.10 (1.07, 1.13) | <.001 |

Multivariable model (1) was adjusted for age, sex, body mass index, income levels, smoking, alcohol consumption, regular physical activity, hypertension, diabetes mellitus, dyslipidemia, stroke, atrial fibrillation, renal disease, cancer, aspartate aminotransferase, and alanine aminotransferase levels.

Multivariable model (2) was adjusted for age, sex, body mass index, income levels, smoking, alcohol consumption, regular physical activity, hypertension, diabetes mellitus, dyslipidemia, stroke, atrial fibrillation, renal disease, cancer, aspartate aminotransferase, alanine aminotransferase, and mean gamma-glutamyl transferase levels.

HR, hazard ratio; CI, confidence interval; Q, quartile.
