# Supplementary material for: Association of gamma-glutamyl transferase variability with risk of osteoporotic fractures: A nationwide cohort study
Source: PLoS One. 2023 Jun 2;18(6):e0277452. doi: 10.1371/journal.pone.0277452 (PMC10237661; doi:10.1371/journal.pone.0277452)
Supplement: S3 Table — (DOCX) [file pone.0277452.s004.docx]

**Supplementary Table 3.** The risk for the occurrence of osteoporotic fractures according to quartiles of gamma-glutamyl transferase variability, landmark analysis using a start time of 1 year after the index date.

|  |  |  |  |  |  | Multivariable model (1) | | | Multivariable model (2) | | |
| --- | --- | --- | --- | --- | --- | --- | --- | --- | --- | --- | --- |
|  | Number of  participants | Number of  events | Event rate (%) (95% CI) | Person-years | Incidence rate (per 1000 person-years) | Adjusted HR (95% CI) | *P*-value | *P*-value for trend | Adjusted HR (95% CI) | *P*-value | *P*-value for trend |
| CV |  |  |  |  |  |  |  | <.001 |  |  | <.001 |
| Q1 | 268078 | 11711 | 4.37 (4.29, 4.45) | 3231920.61 | 3.62 | 1 (reference) |  |  | 1 (reference) |  |  |
| Q2 | 268138 | 11409 | 4.25 (4.18, 4.33) | 3233826.81 | 3.53 | 1.03 (1.00, 1.05) | 0.064 |  | 1.03 (1.00, 1.05) | 0.064 |  |
| Q3 | 268108 | 11830 | 4.41 (4.33, 4.49) | 3224312.11 | 3.67 | 1.07 (1.04, 1.10) | <.001 |  | 1.07 (1.04, 1.10) | <.001 |  |
| Q4 | 268108 | 13667 | 5.10 (5.01, 5.18) | 3190446.44 | 4.28 | 1.15 (1.12, 1.18) | <.001 |  | 1.15 (1.12, 1.18) | <.001 |  |
| SD |  |  |  |  |  |  |  | <.001 |  |  | <.001 |
| Q1 | 268562 | 12771 | 4.76 (4.67, 4.84) | 3240062.35 | 3.94 | 1 (reference) |  |  | 1 (reference) |  |  |
| Q2 | 267563 | 12007 | 4.49 (4.41, 4.57) | 3223647.33 | 3.73 | 1.02 (0.99, 1.05) | 0.127 |  | 1.02 (0.99, 1.04) | 0.234 |  |
| Q3 | 268213 | 11538 | 4.30 (4.22, 4.38) | 3225836.35 | 3.58 | 1.08 (1.05, 1.11) | <.001 |  | 1.06 (1.03, 1.09) | <.001 |  |
| Q4 | 268094 | 12301 | 4.59 (4.51, 4.67) | 3190959.96 | 3.86 | 1.22 (1.19, 1.25) | <.001 |  | 1.11 (1.08, 1.15) | <.001 |  |
| VIM |  |  |  |  |  |  |  | <.001 |  |  | <.001 |
| Q1 | 268108 | 11585 | 4.32 (4.24, 4.40) | 3229724.34 | 3.59 | 1 (reference) |  |  | 1 (reference) |  |  |
| Q2 | 268119 | 11214 | 4.18 (4.11, 4.26) | 3232381.24 | 3.47 | 1.02 (0.99, 1.04) | 0.234 |  | 1.01 (0.99, 1.04) | 0.337 |  |
| Q3 | 268136 | 11807 | 4.40 (4.32, 4.48) | 3224471.85 | 3.66 | 1.06 (1.03, 1.09) | <.001 |  | 1.05 (1.02, 1.08) | <.001 |  |
| Q4 | 268069 | 14011 | 5.23 (5.14, 5.31) | 3193928.55 | 4.39 | 1.12 (1.09, 1.15) | <.001 |  | 1.10 (1.07, 1.13) | <.001 |  |

Multivariable model (1) was adjusted for age, sex, body mass index, income levels, smoking, alcohol consumption, regular physical activity, hypertension, diabetes mellitus, dyslipidemia, stroke, atrial fibrillation, renal disease, cancer, aspartate aminotransferase, and alanine aminotransferase levels.

Multivariable model (2) was adjusted for age, sex, body mass index, income levels, smoking, alcohol consumption, regular physical activity, hypertension, diabetes mellitus, dyslipidemia, stroke, atrial fibrillation, renal disease, cancer, aspartate aminotransferase, alanine aminotransferase, and mean gamma-glutamyl transferase levels.

CI, confidence interval; HR, hazard ratio; CV, coefficient of variation; Q, quartile; SD, standard deviation; VIM, variability independent of the mean.
