# Supplementary material for: Association of gamma-glutamyl transferase variability with risk of osteoporotic fractures: A nationwide cohort study
Source: PLoS One. 2023 Jun 2;18(6):e0277452. doi: 10.1371/journal.pone.0277452 (PMC10237661; doi:10.1371/journal.pone.0277452)
Supplement: S4 Table — (DOCX) [file pone.0277452.s005.docx]

**Supplementary Table 4.** The subgroup analysis regarding gamma-glutamyl transferase variability based on the coefficient of variation and osteoporotic fractures in association with demographics or comorbidities.

| Variable | Adjusted HR (95% CI) | *P*-value for interaction effect |
| --- | --- | --- |
| Age, years |  | 0.052 |
| <65 |  |  |
| Q1 | 1 (reference) |  |
| Q2 | 1.01 (0.98,1.04) |  |
| Q3 | 1.04 (1.02,1.07) |  |
| Q4 | 1.13 (1.10,1.16) |  |
| ≥65 |  |  |
| Q1 | 1 (reference) |  |
| Q2 | 1.02 (0.97,1.07) |  |
| Q3 | 1.05 (1.02,1.08) |  |
| Q4 | 1.08 (1.06,1.10) |  |
| Sex |  | 0.035 |
| Male |  |  |
| Q1 | 1 (reference) |  |
| Q2 | 1.01 (0.97,1.05) |  |
| Q3 | 1.04 (1.00,1.08) |  |
| Q4 | 1.08 (1.04,1.12) |  |
| Female |  |  |
| Q1 | 1 (reference) |  |
| Q2 | 1.03 (1.00,1.07) |  |
| Q3 | 1.09 (1.05,1.13) |  |
| Q4 | 1.19 (1.15,1.23) |  |
| Body mass index (kg/m^2^) |  | 0.533 |
| <25 |  |  |
| Q1 | 1 (reference) |  |
| Q2 | 1.02 (0.98,1.05) |  |
| Q3 | 1.07 (1.04,1.11) |  |
| Q4 | 1.14 (1.11,1.18) |  |
| ≥25 |  |  |
| Q1 | 1 (reference) |  |
| Q2 | 1.03 (0.98,1.08) |  |
| Q3 | 1.05 (1.00,1.10) |  |
| Q4 | 1.14 (1.09,1.19) |  |
| Household income |  | 0.376 |
| Q1, lowest |  |  |
| Q1 | 1 (reference) |  |
| Q2 | 1.01 (0.96,1.06) |  |
| Q3 | 1.05 (1.00,1.11) |  |
| Q4 | 1.10 (1.05,1.15) |  |
| Q2 |  |  |
| Q1 | 1 (reference) |  |
| Q2 | 1.01 (0.96,1.05) |  |
| Q3 | 1.07 (1.02,1.12) |  |
| Q4 | 1.16 (1.11,1.21) |  |
| Q3 |  |  |
| Q1 | 1 (reference) |  |
| Q2 | 1.03 (0.99,1.09) |  |
| Q3 | 1.06 (1.01,1.11) |  |
| Q4 | 1.17 (1.11,1.22) |  |
| Q4, highest |  |  |
| Q1 | 1 (reference) |  |
| Q2 | 1.05 (0.99,1.13) |  |
| Q3 | 1.11 (1.04,1.18) |  |
| Q4 | 1.17 (1.09,1.25) |  |
| Smoking |  | 0.058 |
| Never |  |  |
| Q1 | 1 (reference) |  |
| Q2 | 1.01 (0.98,1.05) |  |
| Q3 | 1.06 (1.02,1.09) |  |
| Q4 | 1.11 (1.08,1.14) |  |
| Former |  |  |
| Q1 | 1 (reference) |  |
| Q2 | 1.00 (0.93,1.09) |  |
| Q3 | 1.06 (0.98,1.14) |  |
| Q4 | 1.19 (1.11,1.29) |  |
| Current |  |  |
| Q1 | 1 (reference) |  |
| Q2 | 1.06 (1.00,1.12) |  |
| Q3 | 1.11 (1.05,1.16) |  |
| Q4 | 1.21 (1.15,1.28) |  |
| Alcohol consumption (days/week) |  | 0.027 |
| <1 |  |  |
| Q1 | 1 (reference) |  |
| Q2 | 1.01 (0.98,1.04) |  |
| Q3 | 1.05 (1.02,1.08) |  |
| Q4 | 1.11 (1.08,1.15) |  |
| 1-4 |  |  |
| Q1 | 1 (reference) |  |
| Q2 | 1.07 (1.01,1.12) |  |
| Q3 | 1.13 (1.08,1.19) |  |
| Q4 | 1.22 (1.16,1.28) |  |
| ≥5 |  |  |
| Q1 | 1 (reference) |  |
| Q2 | 1.10 (0.92,1.33) |  |
| Q3 | 1.13 (0.94,1.35) |  |
| Q4 | 1.28 (1.09,1.51) |  |
| Regular physical activity (days/week) |  | 0.095 |
| <1 |  |  |
| Q1 | 1 (reference) |  |
| Q2 | 1.02 (0.98,1.06) |  |
| Q3 | 1.08 (1.04,1.12) |  |
| Q4 | 1.17 (1.13,1.21) |  |
| 1-4 |  |  |
| Q1 | 1 (reference) |  |
| Q2 | 1.02 (0.98,1.06) |  |
| Q3 | 1.07 (1.03,1.11) |  |
| Q4 | 1.13 (1.09,1.17) |  |
| ≥5 |  |  |
| Q1 | 1 (reference) |  |
| Q2 | 1.06 (0.97,1.16) |  |
| Q3 | 1.00 (0.92,1.10) |  |
| Q4 | 1.12 (1.02,1.22) |  |
| Comorbidities |  |  |
| Hypertension |  | 0.260 |
| No |  |  |
| Q1 | 1 (reference) |  |
| Q2 | 1.02 (0.99,1.06) |  |
| Q3 | 1.07 (1.04,1.11) |  |
| Q4 | 1.15 (1.12,1.19) |  |
| Yes |  |  |
| Q1 | 1 (reference) |  |
| Q2 | 1.01 (0.97,1.06) |  |
| Q3 | 1.05 (1.00,1.10) |  |
| Q4 | 1.13 (1.08,1.18) |  |
| Diabetes mellitus |  | 0.668 |
| No |  |  |
| Q1 | 1 (reference) |  |
| Q2 | 1.02 (1.00,1.05) |  |
| Q3 | 1.07 (1.04,1.10) |  |
| Q4 | 1.15 (1.12,1.18) |  |
| Yes |  |  |
| Q1 | 1 (reference) |  |
| Q2 | 1.01 (0.94,1.08) |  |
| Q3 | 1.04 (0.97,1.11) |  |
| Q4 | 1.13 (1.07,1.21) |  |
| Dyslipidemia |  | 0.211 |
| No |  |  |
| Q1 | 1 (reference) |  |
| Q2 | 1.02 (0.99,1.05) |  |
| Q3 | 1.08 (1.04,1.11) |  |
| Q4 | 1.16 (1.13,1.19) |  |
| Yes |  |  |
| Q1 | 1 (reference) |  |
| Q2 | 1.01 (0.96,1.07) |  |
| Q3 | 1.05 (1.00,1.10) |  |
| Q4 | 1.12 (1.06,1.17) |  |
| Stroke |  | 0.092 |
| No |  |  |
| Q1 | 1 (reference) |  |
| Q2 | 1.02 (0.99,1.04) |  |
| Q3 | 1.07 (1.04,1.10) |  |
| Q4 | 1.15 (1.12,1.18) |  |
| Yes |  |  |
| Q1 | 1 (reference) |  |
| Q2 | 1.22 (1.03,1.43) |  |
| Q3 | 1.05 (0.88,1.24) |  |
| Q4 | 1.22 (1.05,1.42) |  |
| Atrial fibrillation |  | 0.822 |
| No |  |  |
| Q1 | 1 (reference) |  |
| Q2 | 1.02 (1.00,1.05) |  |
| Q3 | 1.07 (1.04,1.10) |  |
| Q4 | 1.15 (1.12,1.18) |  |
| Yes |  |  |
| Q1 | 1 (reference) |  |
| Q2 | 1.02 (0.74,1.41) |  |
| Q3 | 1.12 (0.82,1.52) |  |
| Q4 | 1.05 (0.80,1.39) |  |
| Renal disease |  | 0.897 |
| No |  |  |
| Q1 | 1 (reference) |  |
| Q2 | 1.02 (1.00,1.05) |  |
| Q3 | 1.07 (1.04,1.10) |  |
| Q4 | 1.15 (1.12,1.18) |  |
| Yes |  |  |
| Q1 | 1 (reference) |  |
| Q2 | 0.99 (0.83,1.19) |  |
| Q3 | 1.03 (0.87,1.23) |  |
| Q4 | 1.11 (0.94,1.31) |  |
| Cancer |  | 0.296 |
| No |  |  |
| Q1 | 1 (reference) |  |
| Q2 | 1.02 (0.99,1.05) |  |
| Q3 | 1.07 (1.04,1.09) |  |
| Q4 | 1.14 (1.11,1.17) |  |
| Yes |  |  |
| Q1 | 1 (reference) |  |
| Q2 | 1.12 (0.97,1.28) |  |
| Q3 | 1.16 (1.01,1.32) |  |
| Q4 | 1.30 (1.15,1.48) |  |

HR, hazard ratio; CI, confidence interval; Q, quartile.
