# Supplementary material for: Association of gamma-glutamyl transferase variability with risk of osteoporotic fractures: A nationwide cohort study
Source: PLoS One. 2023 Jun 2;18(6):e0277452. doi: 10.1371/journal.pone.0277452 (PMC10237661; doi:10.1371/journal.pone.0277452)
Supplement: S8 Table — (DOCX) [file pone.0277452.s009.docx]

**Supplementary Table 8.** The risk for the occurrence of humerus fractures according to quartiles of gamma-glutamyl transferase variability.

|  | Number of participants | Number of events | Event rate (%) (95% CI) | Person-years | Incidence rate (per 1000 person-years) | Adjusted HR (95% CI) | *P*-value | *P*-value for trend |
| --- | --- | --- | --- | --- | --- | --- | --- | --- |
| CV |  |  |  |  |  |  |  | <.001 |
| Q1 | 268078 | 838 | 0.31 (0.29, 0.33) | 3287060.61 | 0.26 | 1 (reference) |  |  |
| Q2 | 268138 | 889 | 0.33 (0.31, 0.35) | 3286902.04 | 0.27 | 1.07 (0.97, 1.17) | 0.172 |  |
| Q3 | 268108 | 991 | 0.37 (0.35, 0.39) | 3278869.50 | 0.30 | 1.18 (1.08, 1.29) | <.001 |  |
| Q4 | 268108 | 1192 | 0.44 (0.42, 0.47) | 3251038.74 | 0.37 | 1.36 (1.24, 1.48) | <.001 |  |
| SD |  |  |  |  |  |  |  | <.001 |
| Q1 | 268562 | 874 | 0.33 (0.30, 0.35) | 3301018.62 | 0.27 | 1 (reference) |  |  |
| Q2 | 267563 | 866 | 0.32 (0.30, 0.35) | 3280460.83 | 0.26 | 1.04 (0.95, 1.15) | 0.407 |  |
| Q3 | 268213 | 956 | 0.36 (0.33, 0.38) | 3278634.14 | 0.29 | 1.18 (1.07, 1.30) | 0.001 |  |
| Q4 | 268094 | 1214 | 0.45 (0.43, 0.48) | 3243757.29 | 0.37 | 1.48 (1.34, 1.64) | <.001 |  |
| VIM |  |  |  |  |  |  |  | <.001 |
| Q1 | 268108 | 855 | 0.32 (0.30, 0.34) | 3283829.39 | 0.26 | 1 (reference) |  |  |
| Q2 | 268119 | 905 | 0.34 (0.32, 0.36) | 3284182.54 | 0.28 | 1.07 (0.97, 1.18) | 0.159 |  |
| Q3 | 268136 | 995 | 0.37 (0.35, 0.39) | 3278748.36 | 0.30 | 1.17 (1.07, 1.28) | 0.001 |  |
| Q4 | 268069 | 1155 | 0.43 (0.41, 0.46) | 3257110.60 | 0.36 | 1.28 (1.18, 1.40) | <.001 |  |

Multivariable model was adjusted for age, sex, body mass index, income levels, smoking, alcohol consumption, regular physical activity, hypertension, diabetes mellitus, dyslipidemia, stroke, atrial fibrillation, renal disease, cancer, aspartate aminotransferase, alanine aminotransferase, and mean gamma-glutamyl transferase levels.

CI, confidence interval; HR, hazard ratio; CV, coefficient of variation; SD, standard deviation; VIM, variability independent of the mean.
